# The Interplay of English as a Foreign Language Learners’ Interest, Self-Efficacy, and Involvement

**DOI:** 10.3389/fpsyg.2022.837286

**Published:** 2022-03-18

**Authors:** Zhengyuan Liu

**Affiliations:** Foreign Languages School, Huanghuai University, Zhumadian, China

**Keywords:** academic interest, self-efficacy, students’ involvement, learners’ success, language education

## Abstract

Founded on the advent of Positive Psychology in recent decades, the learners’ involvement has been a critical issue since the origin of teaching and learning despite it being quickly developed in previous decades. The enhancement of motivational aspects like self-efficacy and interest appears to have a high impact on learners’ success and achievement. Although both constructs are extensively investigated in various subjects, their association between and the learners’ involvement in the process of language learning have not been taken into account so far. This review intended to scrutinize the association among students’ self-efficacy, their academic interest, and their involvement. It is significant to pinpoint that the current review can help educational administrations, professional improvement centers, and policymakers to consider the above-mentioned issues in the progression of language education to develop their involvement.

## Introduction

Students ought to actively exercise and utilize language over a prolonged duration of time to increase their development in foreign language education. Consequently, a central principle of communicative methods puts students’ involvement at the core of effective language education. It focuses on the students’ participation in meaningful language communication, active use, and utilization of the language (Mercer and Dörnyei, 2020). Teachers of all levels, from the beginning level up to higher levels, are permanently worried about learners’ involvement on the one hand, and their success on the other hand. Hence, they often think about how some learners are involved, engaged, and interested in coursework while others are neither involved nor interested, despite being in the same teaching space (Linnenbrink and Pintrich, 2003). As stated by Mercer (2019), education calls for active involvement on the student’s part, and stroke is the describing attribute of student engagement. In the ordinary sense, engagement has a wider implication associated with being immersed or occupied in conducting something inside the domain of instruction and education. However, involvement goes beyond the issue and alludes to the amount and excellence of students’ dynamic involvement and engrossment in a language education assignment or activity. An involved student is enthusiastically engaged in and dedicated to their education, and a significant education is improbable without engagement (Wang et al., 2021). The developing acknowledgment for the significance of engagement in present-day learning has become one of the most famous research topics in learning, to the point that it had been defined as the sanctified grail of education (Sinatra et al., 2015).

Grounded on the evidence that constructive feelings are encouraging to involvement and language education, some peripheral, noticeable, and action-oriented manners exist. These are signs of constructive emotions, e.g., enjoyment, interest, confidence, and attentiveness (Reschly et al., 2008). One of the main constructive feelings which encourage investigation and problem resolving during learning is the feeling of interest (Izard, 2009). Interest is regarded as the most central and widespread human feeling which directs awareness and helpfulness. In addition, it is of specific prominence in early improvement as learners are fascinated by how things and individuals are and how they are inclined to discover and encounter the setting (Renninger, 2000). Interest has a dynamic function in learning. In second or foreign language education, it is predominantly referred to be an intensified thoughtfulness and an emotive commitment that arises once an individual has an optimistic communication with content or activity (Hidi and Renninger, 2006). To educators, a learner is interested and involved in the teaching space when they show their interest in assignments, feel enthusiastic about it, or consider it as significant and valuable. Enthusiasm investigation has revealed that these outlooks and principles about interest as emotions bring about more involvement and success as an attentive and happy person engrosses new facts and practices more enthusiastically, thereby causing the development of one’s identity (Linnenbrink and Pintrich, 2003).

In addition, in the last two periods, positive features like self-efficacy have been looked upon as one of the greatest predictors of accomplishment in learners. In line with this, an individual’s self-efficacy has been described to envisage educational accomplishments (Chemers et al., 2001). Regarding engagement, self-efficacy is significant at several phases of education, and it is an effective sign of scholastic presentation, educational achievement, and many other significant elements (Feldman and Kubota, 2015). For instance, self-efficacy is about regulating what activities students choose to be involved in along with their ongoing engagement to persevering and finalizing assignments or activities (Mercer, 2019). Self-efficacy is crucial to the conception of assistance, in which people are vigorously involved in their lives and prospects. These principles have been associated with an array of the significant contributing factors of educational achievement, including enthusiasm, perseverance, and self-regulation (Doménech-Betoret et al., 2017). Motivational scholars have maintained that self-efficacy, well-defined as learners’ philosophies in their competency to study a prearranged issue or efficaciously do a precise task, is a strong indicator of commitment (Bandura, 2011). It is vital to increase this intellect of self-efficacy and a learner’s prospect for achievement. In addition, their emotional state toward their capability can be very thoroughly correlated with their interest to acquire or involve in more demanding activities (Malureanu et al., 2021).

As declared by Baker et al. (2010), fatigue and exhaustion due to the absence of interest are characterized as boredom. Such fatigue and shortage of involvement suggest a lack of emotional energy for education. This type of boredom could also jeopardize intellectual engagement by decreasing intellectual resources and subverting both intrinsic and extrinsic values so the role of interest in this domain is significant (Pekrun, 2011). The construct of self-efficacy might also be interrelated to commitment as it results in readiness to spend supplementary vigor and energy on carrying out tasks or activities, thereby increasing task participation and engagement (Ouweneel et al., 2011). Even though interest has been generally investigated in several educational disciplines, it has mostly been ignored in the field of English language education (Tin, 2016). While interest, also defined as being involved in a studying activity for joy, is one of the determinants that could affect learners’ learning achievements, it can be one of the most overlooked elements in classroom environments (Bai et al., 2020). Educators can also focus more on learners’ real competence than on learners’ self-perceived competence in finishing a particular assignment, that is, self-efficacy (Chong, 2007). Other field studies have also used correlational design to prove a constructive relation between self-efficacy and involvement (Llorens et al., 2007; Ouweneel et al., 2011). As a matter of fact, as was demonstrated in an intervention research among learners, altered transformations in degrees of self-efficacy are linked to parallel transformations in degrees of vitality and devotion (Bresó et al., 2011). In addition, there are numerous inquiries on self-efficacy, interest, and engagement (Chemers et al., 2001; Fredricks et al., 2016; Sökmen, 2021). However, based on researchers’ knowledge, there is a dearth of review of literature that has investigated all these three constructs altogether in language learning.

## Review of the Literature

### Students’ Involvement

During class instruction and education, students’ involvement is highly crucial and is one of the deciding elements to estimate students’ achievement (Webber et al., 2013). Students’ involvement is characterized as constructive energy in the shape of the physical and mental dimension of students to attain scholastic experience as part of class instruction and education. Educational involvement could build an advantageous class ambiance and additionally offer a possibility for the students to discover their perspectives (Kuh et al., 2011). An enormous attempt must be made to promote students’ educational involvement, which includes enhancing the quality of instruction, educators’ function, and educational tasks. The advancement of learners’ education which is concerned with the cycle of instruction and educational activity additionally impacts students’ retention and will ultimately affect educational results and students’ quality (Roberts and McNeese, 2010; Wang and Guan, 2020). Fletcher (2003) mentioned six factors with regard to students’ involvement. First, educational involvement ought to be capable of building students’ intricate abilities. Second, it should develop reciprocated partnership between educators and students. Third, equity or students’ involvement ought to contribute similarly among students without any bias toward race, religion, gender, position, financial, and other differential factors. Fourth, engagement must alter students’ educational demeanor in exhaustive and coherent ways. Fifth, the involvement quality ought to illuminate crucial problems. Finally, involvement is a clear guide and measure and satisfies regular requirements of involvement.

### Interest

The expectancy-value theory emphasizes the significance of interest in upgrading the eminence of inspiration in education. The more learners experience joy when performing an assignment, the more they will engage in education, inspiration, and upgrading superficial knowledge processing (Mazer, 2017). Some scholars (e.g., Renninger, 2000; Schraw and Lehman, 2001) in scholastic inquiries have principally concentrated on different kinds of interest. For instance, situational interest is considered as the attentive helpfulness and the emotional response prompted in the second by ecological provocations that may or may not complete over time. Such type of interest is likely to be context-based and of temporary rate. Another kind of interest, namely, the individual interest, talks about one’s comparatively durable disposition to be involved in specific content (Renninger, 2000). Individual interest is theorized as a moderately persistent disposition to be present at a certain task and is related to constructive emotions, determination, and education (Ainley et al., 2002). This type of interest is supposed to be subject-detailed and have enduring individual significance that is interconnected to an individual’s prior information and involvements. Another kind of interest, identified as the topic interest, is presented as what is supposed to be a student’s degree of interest when a particular topic is examined (Ainley et al., 2002). Moreover, academic interests are proposed to be personalities based on psychological representations connecting the matters of awareness with constructive involvements and individual value systems which are triggered in the structure of interest-driven activities. However, a hypothetical difference exists between the value and commitment mechanisms of interest which are not possible to be empirically differentiated between these constituents (Koller et al., 2001).

### Self-Efficacy

Self-efficacy is a foremost subject in describing in what way a person acts, executes, reflects, and responds in the face of stimulating situations that are fundamental in developing students’ manners to abridge their learning development (Downes et al., 2017). People are not automatically controlled by external events and are not stimulated by internal pressures (Bandura, 2011). Instead, they actively participate in what affects their growth. They adapt to change and gradually start self-reestablishment. Bandura’s Social Cognitive Theory (SCT) offers the conceptual reasoning for the assumption that self-efficacy influences scholastic achievement by elevating learners’ feeling of health and the degree of perseverance they portray to conquer difficult scholastic assignments, thereby leading to more effective utilization of attained information and abilities (Bandura, 2011). Thus, the theory enables teachers to understand their behaviors and beliefs, providing them with the understanding of their own beliefs about educational competence (Bandura, 2011). Bandura’s theory includes a conceptual framework encompassing the origins or roots of efficacy beliefs, forms and roles, cycles leading to different influences, and change potential (Brouwers and Tomic, 2000). Self-efficacy is related to the sense of self-reassurance in a person’s aptitude and can be acknowledged as the interest to acquire more or the desire to take part in coursework that is viewed as obstacles, contrary to tasks of wide-ranging attentiveness (Han and Wang, 2021). Self-efficacious students are referred to as those students who expand their abilities and are encouraged to be involved in education. However, the commitment and enthusiasm of the students with low degrees of self-efficacy are minor since self-efficacy has a vivacious function in all facets of educational commitment and involvement (Olivier et al., 2019). Self-efficacy is a concept that stems from someone’s will and belief. It is also derived from SCT and depends on the self-awareness of one’s abilities rather than the actual capacity level (which is a commonly discussed topic) that describes how an individual performs, thinks, and responds to an inspiring situation (Tschannen-Moran and Hoy, 2007). Self-efficacious learners are prone to develop and/or try different behaviors if they fail at their first attempt. Increased degrees of endeavor and perseverance improve classroom presentation, and learners better address difficult circumstances by altering intellectual and emotive cycles associated with those circumstances (Bandura, 2011). Mental conditions, in addition to proficiency experience, which is associated with previous achievements in equivalent assignments, are one of the most significant origins of efficacy convictions. Therefore, the less the stress, worry, and exhaustion, the greater the self-efficacy. If learners have deconstructive thoughts or stress about their abilities, these deconstructive emotional responses further reduce their awareness of their abilities and trigger worry-inducing mechanisms that increase the chances of the insufficient presentation that they dread. For example, learners who are afraid to speak in front of a large audience are generally less confident in their ability to speak in public, thereby resulting in poor presentation, which wrongly gives grounds for and strengthens deconstructive self-efficacy convictions (Bresó et al., 2011).

## Final Remarks

Constructive theoretical feelings like interest and efficacy can support and assist learners to dynamically discover educational occasions and prospects and boost more perseverance and energy. This makes learners feel more committed to the procedure and practice of learning (Reschly et al., 2008). Learner’s interest in foreign language education contexts is an intricate interaction between different characteristics of the user (learner), the object (language-centric material, non-language-centric material, activities), and the context (in-class and out-of-class situational attributes). The addition of interest to learners’ education and success is a leading problem in modern educational studies. Recently, Thoman et al. (2011) demonstrated that the function of interest in education is to use the assets required to accomplish an assignment. If a learner is carrying out an assignment and his or her intellectual assets are exhausted, presenting something intriguing can restore his/her intellectual assets and enhance the probability that the learner will be involved again with new excitement. Interest can be combined with self-efficacy as associated concepts that envisage students’ involvement. Based on the review of literature, the valuable and adaptive function of self-efficacy in the educational field is assured. This conclusion proposes that learners with more self-efficacy are more inclined to enthusiastically and cognitively take part and be involved in the classroom. Indeed, a vital component of career involvement is instructor efficacy as it pertains to the individual skills and abilities that can be used by teachers in any school or academic environment. For instance, elevated self-efficacy results in elevated involvement. This means that learners remain motivated to endeavor in their learning. As mentioned, interest is the best teacher for learners in their learning practice. In the study author’s own teaching context, for example, the author sometimes had to teach struggling students who had a weak proficiency in English. Due to their low English level, they took little interest in class activities. For this group of students, the author would like to design some class activities or use some teaching materials which are closely associated with their daily life. That is to say, always creating some teaching scenarios where they are more willing to get involved in. This has been confirmed by the author’s own teaching experiences and has gained satisfactory learning results. With reference to the constructive function of self-efficacy and interest, higher education faculty members should try to cultivate and present programs that can develop learners’ involvement in the educational spaces. Also, it can be postulated that involvement is viewed as a value of interest. In other words, as people become more interested in their learning, their behavior can reflect their interest when they are involved in different tasks.

The review of the literature infers that learner’s self-efficacy is a basis for better commitment and, accordingly, it has noteworthy values for higher education administrators. As a result, they are inclined to make out learners’ self-efficacy as a vivacious educational source in developing and employing operative and well-organized instructive guidelines that enlighten learners’ maintainable self-efficacy. Efficacious learners are more prone to adjust their inspiration by having their own objectives and (Diseth, 2011) are, thus, more prone to be involved. A fundamental instructional implication taken from this mini-review is that educators should search for ways to develop an interest in language learning and should employ the foreign language in class to the extent that nurtures not only interest and satisfaction, but also sustains and triggers engagement in the classroom. As an important motivational variable, interest can cause a higher degree of self-regulation and self-efficacy (Tin, 2016). Self-efficacy and interest could change learners’ English learning while the assignments are very difficult. Still, learners can meta-cognitively and successfully modify their English learning. Moreover, the learners with a better degree of self-efficacy and interest are willing to be engaged in learners than learners with a smaller degree of self-efficacy and interest. Academic self-efficacy and interest, together, mitigate the effect of assignment significance on learning behavior (Mazer, 2017). This is because interest has a beneficial effect on learners’ learning and self-efficacy and is proved to be a strong motivation-academic achievement predictor (Komarraju and Dial, 2014; McIlroy et al., 2015). According to Bandura (2011), learners with high self-efficacy are willing to be more inclined to participate, overcome difficulties, and put more effort into achieving goals, while learners with low self-efficacy have a tendency to cling to past mistakes and reduce effort on challenging tasks. Learners’ perception of the self-efficacy of their learning plays an important role in motivating and engaging learners (Linnenbrink and Pintrich, 2003). The inability to build interest in youths could result in great degrees of boredom, which is not favorable for health. During youth, maintained interest enhances the grownup’s devotion to attaining knowledge, which leads to their engagement and life-long education (Ely et al., 2013).

Learners with a greater level of educational self-efficacy have been considered to accomplish both challenging school responsibilities and their school time management in a better manner (Doménech-Betoret et al., 2017). The explanation provided by an individual for his or her achievements is called self-efficacy, which is generated by current self-convictions that directly affect the individual’s inspiration to achieve success in future situations. Thus, teacher training programs can be designed to constructively enhance teachers’ awareness of probable inefficiencies and higher levels of teacher self-efficacy. It has been concluded that learners with a low level of educational self-efficacy are more helpless and less eager to accomplish theoretical responsibilities. Students with a low level of self-efficacy deem themselves unsuccessful. They think about failure circumstances that challenge presentation by concentrating on those capabilities they do not enjoy and often envisaging the worst consequences. Notably, this way of thinking is disadvantageous to a learner and affects their involvement. This may be related to the motivational purpose of educational self-efficacy which talks about individuals’ self-confidence in their aptitudes to complete specific educational responsibilities. Particularly, this motivational purpose can be reflected as a motivational vigor. Learners who encounter and practice more sense of self-efficacy are observed to be interested in using further techniques to expand intellectual ability. They are also ones to put most effort and be involved in the class.

This review provides some evidence that self-efficacy and interest may be related to learners’ involvement. Hence, first and foremost, it recommends future investigations to examine these two characteristics for both teachers and learners. Researchers are recommended to carry out a more experimental search regarding the mutual relations among English as a foreign language (EFL) learners’ self-efficacy, interest, and involvement to detect these upshots in different situations. Second, forthcoming investigations can be done in this domain by taking into account the above variables to comprehend and arrange perceptions for a better theoretical atmosphere. This may be a successful study in this realm, and it will prove to be a substantial support in the progression of learning especially in language learning.

## Author Contributions

The author confirms being the sole author of the current manuscript and approves its submission to Frontiers in Pscyhology.

## Conflict of Interest

The author declares that the research was conducted in the absence of any commercial or financial relationships that could be construed as a potential conflict of interest.

## Publisher’s Note

All claims expressed in this article are solely those of the authors and do not necessarily represent those of their affiliated organizations, or those of the publisher, the editors and the reviewers. Any product that may be evaluated in this article, or claim that may be made by its manufacturer, is not guaranteed or endorsed by the publisher.
